# Application of co-design to develop and prioritise health literacy-informed action ideas for implementation across prisons in New South Wales, Australia

**DOI:** 10.1038/s41598-025-27531-7

**Published:** 2025-12-10

**Authors:** Scott W. Gill, Julia Bowman, Christina Cheng, Caron Shaw, Grantley Creighton, Gary Nicholls, Wendy Hoey, Colette McGrath, Aarifah Bhatti, Kelly-Anne Stewart, Richard H. Osborne

**Affiliations:** 1 Global Health and Equity Development Hub, Violet Vines Marshman Centre for Rural Health Research, La Trobe Rural Health School, Room W106, Level 1, David Myers Building West, Bundoora, VIC 3083 Australia; 2 Justice Health and Forensic Mental Health Network, Sydney, Australia; 3 School of Health Sciences, Swinburne University of Technology, Melbourne, Australia; 4 Patient Safety Team, Clinical Excellence Commission, Sydney, Australia; 5 Corrective Services New South Wales, Sydney, Australia; 6 Department of Public Health, University of Copenhagen, Kobenhavn, Denmark

**Keywords:** Health literacy development, People in prison, Co-design, Ophelia process, Health literacy, Health equity, Health care, Health policy, Health services, Outcomes research, Translational research

## Abstract

The value of considering health literacy in informing intervention development for diverse populations is well-recognised. People in prison frequently experience vulnerabilities and poor health, making prisons an important health-promoting environment. This study applied the Ophelia (Optimising Health Literacy and Access) process in New South Wales (NSW) prisons in Australia to co-design and prioritise health literacy-informed action ideas (i.e., suggested ideas with the potential to improve the health and equity for individuals or groups of people). In 2024, ideas generation workshops and yarning circles were held across six sites, where participants discussed vignettes and were asked to suggest action ideas to support the persona depicted. Staff then rated identified action ideas on their importance, current implementation and feasibility. Senior leaders then participated in a workshop to prioritise the action ideas. A total of 107 participants (people in prison = 54; staff = 53) discussed 23 diverse vignettes in eighteen ideas generation workshops and three yarning circles. From over one thousand suggestions, 260 action ideas were identified and inductively analysed, resulting in 17 strategies and seven action areas. Sixty-five staff rated the action ideas, informing the prioritisation workshop (attended by 43 senior leaders), where 109 action ideas were prioritised into seven priority areas for intervention development, testing and evaluation. This bottom-up and top-down co-design approach lays a strong foundation for service redesign, quality improvement and future research. Prioritised action ideas will now be developed into interventions and trialled in NSW prisons, which have the potential to improve the equity and well-being of people in prison globally.

## Introduction

Health literacy refers to the ability of individuals and communities to access, understand, appraise, remember, and use health information and services^1^. It plays a crucial role in enabling people to engage with health services and manage their own health and that of others^1–3^. Health literacy development, driven by workers and policymakers, focuses on creating enabling environments that support and strengthen these capabilities^1^. Such approaches have been shown to drive behaviour change and improve health outcomes, particularly among populations experiencing vulnerabilities^4–7^. Globally, people in prison tend to have worse health than their community counterparts^8–10^. Likewise, the prison population in New South Wales (NSW), Australia, experiences a higher disease burden than the general population^10–14^. Moreover, a large cross-sectional health literacy survey (*n* = 471) in 14 NSW prisons recently reported that people in prison have lower health literacy than the general population, as measured using the Health Literacy Questionnaire (HLQ)^15^. Therefore, improving the health literacy of people in prison has the potential to improve the health outcomes of this population.

### Prisons can be key health-promoting environments

It is common for people in prison to have a history of trauma, substance misuse and low engagement with health services before and throughout their incarceration^11,14,16–18^. Moreover, people experiencing such vulnerabilities may have suboptimal interactions with health services, including inflexible service delivery and stigmatising attitudes from health professionals^19–21^. These negative experiences could further discourage engagement with and use of health services. This limited engagement and suboptimal experiences with community-based health services before incarceration makes prison health services a potential platform for promoting health and behaviour change within the prison population^8,22–25^. Therefore, when prison and health authorities provide appropriate care and equip individuals with the necessary knowledge and support, they can improve their health outcomes both during incarceration and upon return to the community.

Under the widely accepted Nelson Mandela Rules, people in prison are entitled to the equivalence of care that would be available in the community^26^. In Australia, the government authorities responsible for providing care to people in prison vary between states and territories. In NSW, Australia’s most populous state of 8.5 million people^27^, there are 34 adult prisons, 31 of which are publicly run^28^. Corrective Services NSW and the Justice Health and Forensic Mental Health Network (Justice Health NSW) closely collaborate to provide care for people in public prisons across NSW. Despite this, prisons present challenges to providing equitable healthcare and implementing interventions. Prisons are designed to restrict an individual’s liberty^29^ and are an environment where security and safety can take precedence over healthcare service delivery^30^. Moreover, systematic reviews have shown that while a range of interventions have been implemented in prisons globally^31–36^, health disparities between people in prison and the general population persist^11,14^. This aligns with recent research showing that many interventions in prison settings lack the necessary evidence and rigour to support sustained improvements in health outcomes for people in prison^22^.

Prisons are also crucial settings for advancing the United Nations 2030 Sustainability Development Goals^37^ given their role in addressing health, inequality, and social justice. Sustainability Goal 3 specifically focuses on ensuring healthy lives and promoting well-being for all people at all stages of life^37^. More specifically, the prison context is key to ending the epidemics for some communicable diseases (3.3) (e.g., Hepatitis C), reducing premature mortality from non-communicable diseases (3.4) and strengthening the prevention and treatment of substance abuse (3.5)^37^. If this goal is to be achieved by 2030, alternative approaches to service provision and intervention development, such as understanding and using health literacy as a mechanism, are important considerations. Notably, such approaches have not previously been applied within prison settings globally.

### Using health literacy development to co-design meaningful action ideas

The World Health Organization (WHO) defines health literacy development as how “health workers, services, systems, organisations and policy makers (across government sectors and through cross-sectoral public policies) build the knowledge, confidence and comfort of individuals, families, groups and communities through enabling environments” [1, Box 2, p. 2]. Through creating enabling environments, people are supported to “access, understand, appraise, remember and use information about health and healthcare” [1, Box 2, p.2] for the health and well-being of individuals and their communities throughout their daily lives^1^. The uptake and implementation of health literacy development has been demonstrated through a series of 16 WHO National Health Literacy Demonstration Projects^7^, EU4Health Joint Action on Heart Disease and Diabetes (JACARDI)^38^ and in various other disease and community contexts globally^5,39–45^. These projects put health literacy development into practice by applying the Ophelia (Optimising Health Literacy and Access) process (as described below)^4,46,47^.

This paper reports on the outcomes of the Ophelia co-design process implemented in NSW prisons, highlighting the development and prioritisation of health literacy-informed action ideas to improve the health of incarcerated individuals. Ideas were co-designed by prison staff and people in prison in workshops and, among Aboriginal and Torres Strait Islander participants, yarning circles (a conversational and storytelling approach^48)^. Vignettes of typical people in prison were used to stimulate in-depth conversations and generate action ideas. Stakeholders were then engaged to rate and prioritise the resulting action ideas.

## Methods

**Fig. 1:**
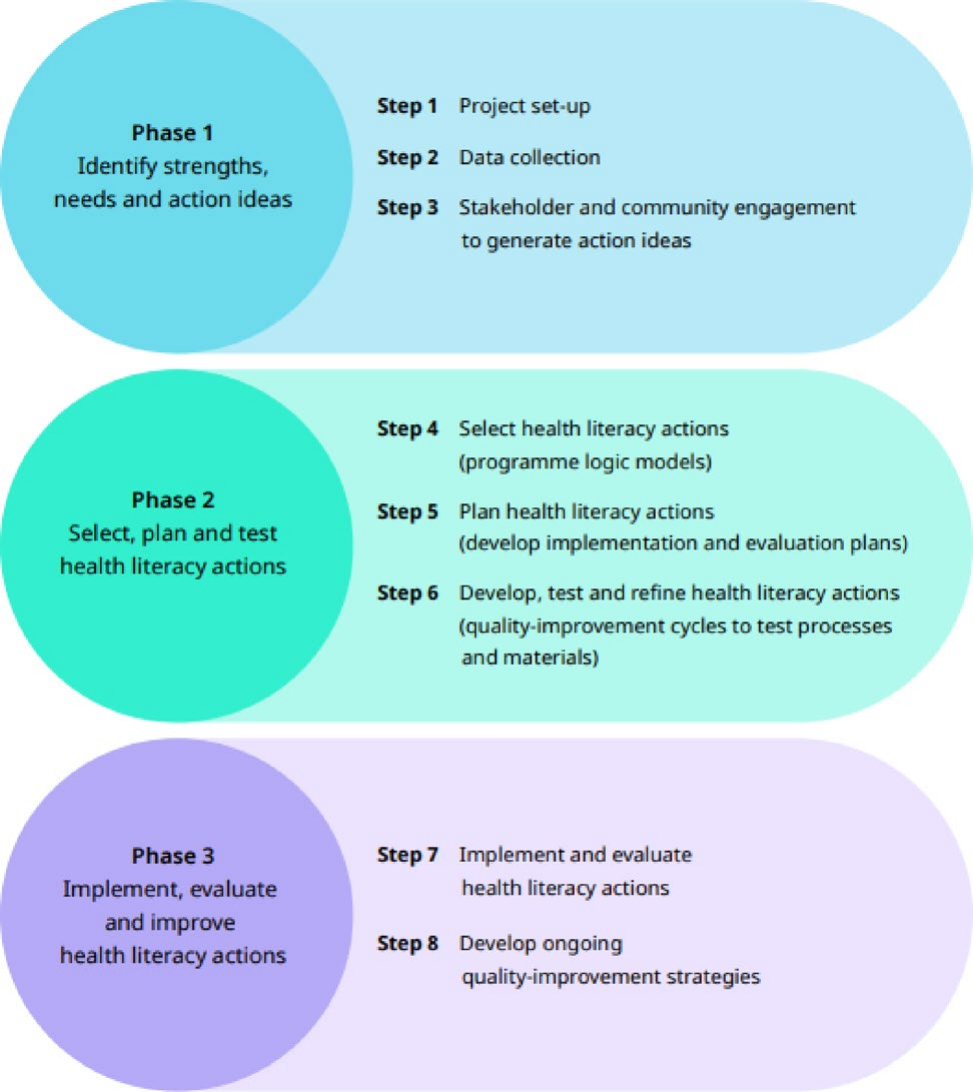
The three phases and eight steps of the Ophelia process. Source: The Ophelia Manual, www.healthliteracydevelopment.com (reproduced with permission)^47^.

The current study was part of a larger program of work undertaken in the Australian prison context^49^ using the Ophelia process^4,46,47^. The Ophelia process is a three-phase, eight-step mixed-methods process that utilises co-design and strength-based approaches (Fig. 1). The Ophelia process included an initial health literacy needs assessment in the population of interest using the 9-scale Health Literacy Questionnaire (HLQ)^50^. Cluster analysis was then applied to the HLQ data to reveal groups of respondents with similar strengths and challenges across the nine health literacy dimensions. Vignettes (stories derived from data) were developed from each cluster profile, using demographic data and insights from semi-structured interviews with the population of interest (e.g., people in prison), exploring their healthcare experiences and reasons for high or low scores on specific HLQ scales. Vignettes were used in ideas generation workshops to stimulate discussions about potential actions and solutions (hereon, action ideas) that could support the needs of the persona depicted. Suggested action ideas were then prioritised in line with the organisation’s and its end-users’ needs (Ophelia Phase 1). Program logic models and an evaluation framework will then be developed (Ophelia Phase 2) to make explicit what prioritised elements are to be tested, implemented and evaluated (Ophelia Phase 3).

The WHO has endorsed the Ophelia process as a methodical way to co-design health literacy-informed action ideas in unique contexts^3,7,51,52^. Throughout the Ophelia process, close collaboration with all stakeholders, including people with lived experience, frontline staff, managers, and executives, has been key to its application and success. This collaboration was undertaken throughout each phase to guide the project, build organisational capacity and ensure the voices of end-users were heard^47^. As the phases and steps of the Ophelia process built upon one another, various stakeholders were involved throughout. Systematically prioritising local leadership, knowledge, and insight supported the development of practical, relevant, and locally tailored health literacy action ideas. Using processes like Ophelia and health literacy development can help create interventions tailored to the unique needs of prison settings and informed by health literacy principles.

**Fig. 2:**
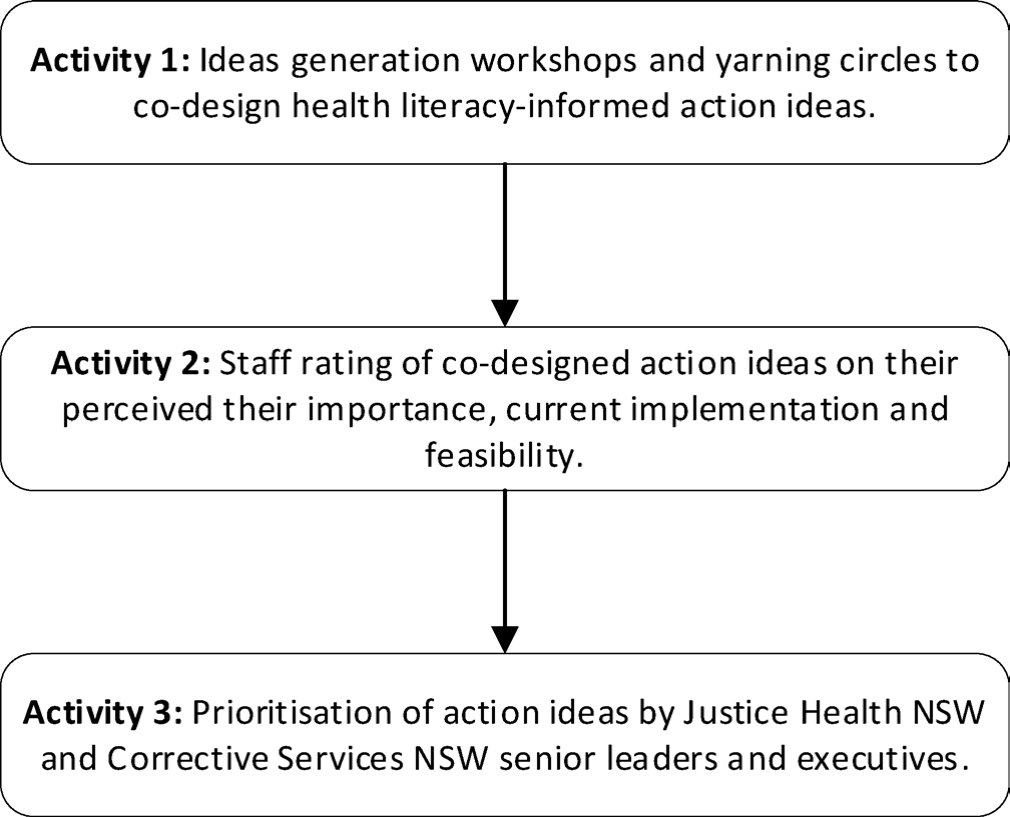
Activities and methods used in Ophelia Phase 1, Step 3.

This paper reports the findings from Phase 1, Step 3 of the Ophelia process: Stakeholder and community engagement to generate action ideas (Fig. 1), using a mixed-methods research design (Fig. 2). It details the use of vignettes in ideas generation workshops and yarning circles to co-design health literacy-informed action ideas (i.e., suggested ideas with the potential to improve health and equity for individuals or groups of people) (Activity 1). These action ideas were rated by staff on their perceived importance, current level of implementation, and feasibility (Activity 2) before being prioritised and grouped into sets of action ideas by senior leaders from Justice Health NSW and Corrective Services NSW (Activity 3).

### Ethics

Ethics approval was obtained from Justice Health and Forensic Mental Health Network Human Research Ethics Committee (HREC) (Ref: 2022/ETH01433), Aboriginal Health and Medical Research Council HREC (Ref: 2007/22), Swinburne University of Technology HREC (Ref: 20236977-15461), Corrective Services Ethics Committee (Ref: D2022/145326), and all methods were performed in accordance with the relevant guidelines and regulations. All participants in the ideas generation workshops and yarning circles provided written informed consent prior to taking part. All discussions from workshops and yarning circles were audio-recorded, and research team members took notes. All audio recordings were transcribed verbatim in preparation for data analysis.

### Activity 1: Ideas generation workshops and yarning circles

#### Participant recruitment

People in prison were recruited to participate via posters in health clinics, information shared at Inmate Development Committees (i.e., a prison community meeting) at each correctional centre, or direct recruitment by the project team and staff within the prison. Direct recruitment of people in prison involved the research team and local prison staff (including Justice Health NSW and Corrective Services NSW) engaging with potential participants at each site, such as in prison health clinics and yards, to promote and share information about the study. People in prison were still required to initiate participation in the study. Justice Health NSW and Corrective Services NSW staff were recruited via email, intranet announcements, posters, and direct recruitment through managers at their respective workplaces. Direct recruitment of staff members involved the research team engaging managers to identify potential participants and to promote and share information about the study. Staff were still required to initiate participation in the study. People in prison were eligible to participate if they were at one of the four correctional centres where workshops or yarning circles were conducted, aged 18 years or older, spoke English and were able to provide written informed consent. Staff eligibility criteria included being a current employee of either Justice Health NSW or Corrective Services NSW, being able to provide written informed consent and having approval from a supervisor or manager to participate.

#### Data collection

Ideas generation workshops and yarning circles took place at four of the 32 publicly run correctional centres and both Justice Health NSW administration sites from April to June 2024. The sites were chosen to capture the diversity in the demographic makeup of people in prison (e.g., males and females), custodial contexts (e.g., differing security classifications) and geographical locations (e.g., rural and metropolitan locations). These factors were considered important to reflect potentially varied needs and perspectives across the NSW prison system.

Twenty of the twenty one workshops and yarning circles were facilitated by the lead author (SWG) with co-facilitation support from at least one other author (JB and CS). One female yarning circle was co-facilitated by authors CS and JB, with support from SWG.

**Fig. 3:**
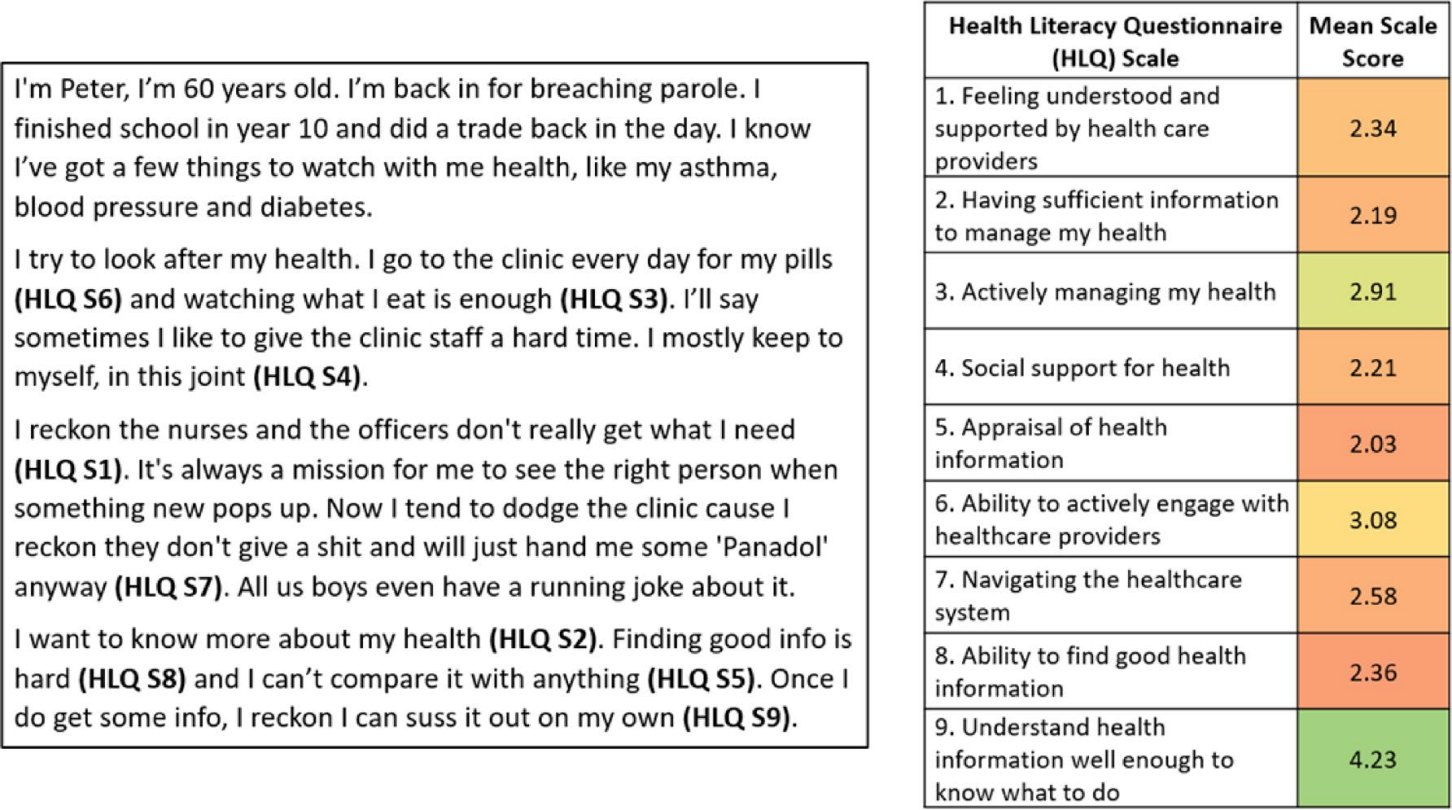
Example vignette with accompanying Health Literacy Questionnaire (HLQ) mean scale scores used during ideas generation workshops and yarning circles.

Note: HLQ S = Health Literacy Questionnaire Scale. HLQ Scales 1–5 are rated on a 4-point agreement scale (1: strongly disagree to 4: strongly agree) and Scales 6–9 are rated on a 5-point ease scale (1: cannot do or always difficult to 5: always easy). HLQ scale numbers (e.g., HLQ S1) were not included in the vignettes presented in the ideas generation workshops and yarning circles. The scale numbers and accompanying scale mean scores are shown here to assist in examining where the vignette text comes from. The colour coding of cells for mean scale scores uses green to indicate the highest levels (e.g., health literacy strengths) and red to indicate the lowest levels (e.g., health literacy challenges), which are relative to the scale scores across clusters.

After obtaining informed consent, facilitators provided an overview of the project, outlining its aims and explaining the structure and process of the ideas generation workshop or yarning circle. For ideas generation workshops, a facilitator read a vignette to the group and then worked through four guiding questions (Table 1). For the yarning circles, the facilitator explained the purpose, read the guiding questions aloud, and then read the vignette to prompt the conversation with the participants. Two to five vignettes, developed in Phase 1, Step 2 of the Ophelia process, were discussed in each workshop or yarning circle, with most having at least three discussed. The initial three workshops used vignettes written in the first and third person. Following advice from the participants, vignettes written in the first person were used thereon. Vignettes were selected to represent individuals who would most likely be incarcerated at the correctional centre or regularly engaging in the services provided by staff at administration sites. For example, when undertaking a workshop at a female prison, vignettes depicting females were presented. Figure 3 provides an example of a vignette.

Slightly different guiding questions were used for the two stakeholder groups (Table 1). The guiding questions differed to accommodate the varying perspectives of staff compared to those of people in prison. The first two questions were used to help the participants develop an understanding of the person represented in the vignette. The remaining two questions guided the discussion around finding local or systemic action ideas.

**Table 1:** Stakeholder group questions to guide ideas generation workshops and yarning circles.

Stakeholder group	Question route
Justice Health NSW and CSNSW staff	1. Do you see people like this person in your centre? 2. What are the issues this person is dealing with? 3. What strategies could be used to help this person? 4. If there were lots of people like this person what could our organisations do to improve outcomes for these people?
People in prison	1. Do you think there are people like this around you in prison? 2. What are the main issues this person is dealing with? 3. What do you think could be done to improve care for this person? 4. What could our organisations do to help people like this person in prison?

Note: Adapted from Gill et al.^49^.

Throughout workshops and yarning circles, facilitators encouraged participants to expand and build upon the action ideas and solutions generated. This included encouraging wider views from other participants. If needed, participants were encouraged to think big, using further prompts such as “In a perfect world, what could be done…” to spur ideas. Facilitators also asked further questions to gain clarity, such as, “So how would that look in practice?” or participants were asked to reframe a barrier and suggest a potential action idea to address it. At the conclusion of each vignette discussion, the co-facilitator summarised the action ideas generated and presented them back to participants to ensure their views were accurately captured (initial participant validation)^53–55^.

#### Data analysis

**Fig. 4:**
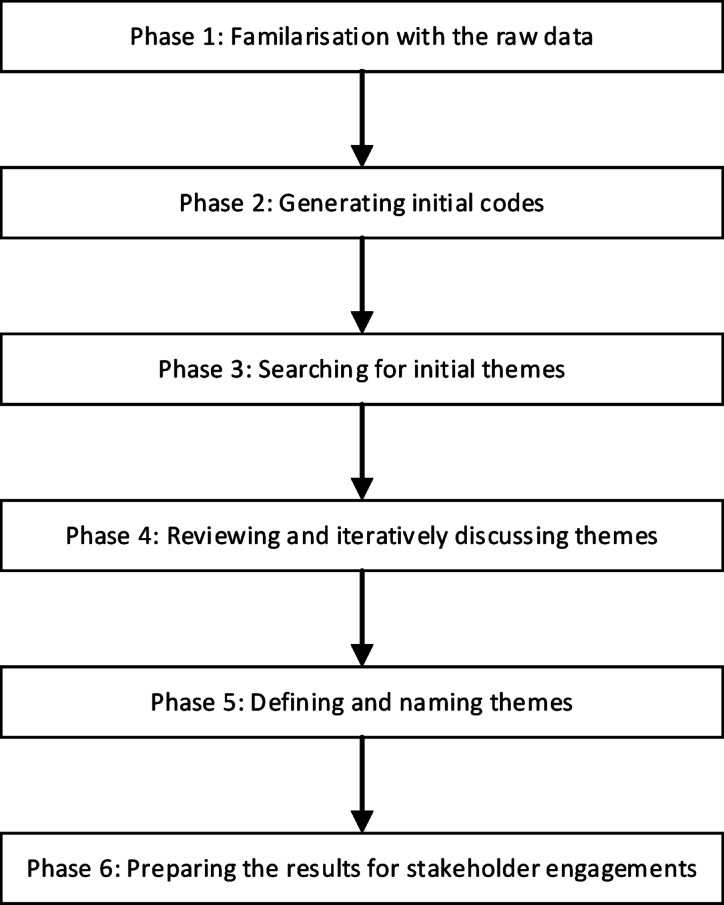
Phases of thematic analysis applied in Ophelia Phase 1, Step 3. Note: Adapted from Braun and Clarke^56^.

An inductive thematic analysis was undertaken following six phases (Fig. 4)^56^. In Phase 1, the lead author (SWG) reviewed transcripts from the ideas generation workshops and yarning circles for familiarisation with the raw data. Potential health literacy-informed action ideas were extracted as line data. Extracted line data were identified by vignette and contextual information (e.g., location, stakeholder group) about the workshop or yarning circle in which they were generated. If more than one potential action idea was identified in a line of extracted data, these were separated. In Phase 2, the lead author reviewed and generated initial codes for the identified potential action ideas. In Phase 3, the coded action ideas were reviewed across all data collection sites to identify patterns and group action ideas into initial themes. During three rounds of review, action ideas were reassigned to more appropriate themes as needed. Initial draft themes were then consolidated across the data collection sites. In Phase 4, a second author (JB) reviewed the draft themes, and iterative discussions were held to define the themes, finalise groupings, and remove duplicates. A third author (RHO) was consulted to resolve any disagreements that had arisen. In Phase 5, each theme was then defined and named to reflect the action ideas grouped within it. In Phase 6, the final action ideas were used to develop a staff rating survey (Activity 2). The action ideas and survey results were compiled into a booklet used by senior leaders and executives in the prioritisation workshop (Activity 3).

### Activity 2: Rating of co-designed action ideas by health and corrections staff

#### Participant recruitment

A total of 205 stakeholders were invited to participate in the survey. Justice Health NSW stakeholders invited to participate included those in clinical roles (e.g., nurses, medical practitioners, allied health professionals), non-clinical roles (e.g., administrative staff), and management positions. From Corrective Services NSW, a diverse range of professional roles were invited, including correctional officers, services and program officers, psychologists, and managers. Invitees included individuals who had previously expressed interest in the project, participated in ideas generation workshops or yarning circles, were members of Justice Health NSW Clinical Councils, or were staff and stakeholders nominated by senior leaders or project team members. While employment in specific professional roles was not a criterion for invitation, this approach ensured broad representation across organisational levels and helped capture diverse perspectives, particularly from often underrepresented groups such as front-line staff.

#### Data collection

An online survey was emailed to stakeholders across Justice Health NSW and Corrective Services NSW. Participants were asked to provide demographic information including organisation (Justice Health NSW, Corrective Services NSW or other), length of time in current role (less than one year, 1–5 years, 6–10 years, 11–15 years, 16–20 years, greater than 20 years), current role (front line staff, non-clinical roles, people manager or middle management, executive manager), Aboriginal Identity (Aboriginal, Torres Strait Islander, both, or neither), culturally and linguistically diverse (CALD) identity (yes or no), and the setting in which they work (metropolitan, rural, administration, male, female, maximum, medium, or minimum security).

To reduce the burden of rating the 260 action ideas (generated in Activity 1), participants were presented with a block of 50 action ideas and asked to rate each on a 5-point scale for their importance (1 = not important at all to 5 = essential), current implementation status (1 = never implemented to 5 = fully implemented) and feasibility (1 = never implemented to 5 = fully implemented). After rating 50 action ideas, participants were invited to continue with up to 3 blocks of 50 action ideas.

#### Data analysis

Survey result data were analysed using IBM SPSS Statistics V27.0^57^. Descriptive analyses were undertaken with frequency and percentages calculated for the 260 action ideas.

### Activity 3: Prioritisation workshop

#### Participant recruitment

Justice Health NSW and Corrective Services NSW senior leaders were invited to attend a three-hour prioritisation workshop. Participants were identified through the Chief Executive of Justice Health NSW and the Acting Commissioner of Corrective Services NSW.

#### Data collection

A prioritisation workshop was conducted with 43 senior leaders and executives from Justice Health NSW and Corrective Services NSW. The workshop’s purpose was for senior leaders to take a broader, strategic perspective on the action ideas generated from workshops and yarning circles and identify higher-level priorities. Participants were presented with an overview of the project and provided with a booklet containing all 260 action ideas (generated in Activity 1) and the results from the rating survey (Activity 2). Participants were divided into six groups, each with staff from various Justice Health NSW streams and Corrective Services NSW. Each group was tasked with reviewing the information provided, identifying connections between individual and grouped action ideas, and prioritising these action ideas or groups of action ideas into four categories: (1) quick wins, (2) within 1 year, (3) within 2–5 years, and (4) over the next 10 years. Participants were encouraged to reorganise action ideas across themes and action areas where they saw connections or overlap. They were also invited to suggest new ideas or refine existing ones. Each group was provided with paper, sticky notes, pens, and markers to record their discussions and prioritisation decisions. After completing the task, groups presented their prioritised action ideas to the larger group. These presentations were audio recorded and transcribed verbatim.

#### Data analysis

Thematic analysis was conducted inductively^56^ in six phases (Fig. 4). In Phase 1, the lead author (SWG) familiarised themselves with the raw data from the workshop transcript. Data were extracted from the transcript for each of the six workshop groups (described above), guided by how participants prioritised action ideas across the four proposed timeframes (i.e., quick wins, within 1 year, within 2–5 years, and over the next 10 years). The extracted data were supplemented with notes recorded by each group on the provided materials, as well as observations and notes from the workshop facilitators. In Phase 2, the data were initially coded for each workshop group across the four prioritisation timeframes. Additional action ideas, suggested by workshop participants but missing from the extracted data, were identified from the booklet and incorporated. In Phase 3, initial themes were developed by searching for patterns across the four timeframes. Initial themes were then synthesised across groups and timeframes, each with a set of associated action ideas. Draft names for each theme were then developed. In Phase 4, a second reviewer (JB) reviewed the action ideas linked to each draft theme. Some action ideas were reassigned if they were better aligned with another theme. The draft themes and associated action ideas were then reviewed by the broader project team. Through an iterative process, the theme names and grouping of action ideas were refined until consensus was reached. In Phase 5, consistent with co-design principles, the project team sought input from the project advisory group to finalise the names of the themes. In Phase 6, the final themes and their associated action ideas were presented to key stakeholders for feedback and validation.

## Results

### Activity 1: Ideas generation workshops and yarning circles

Participants (staff = 53; people in prison = 54) were recruited from across four correctional centre complexes and two Justice Health NSW administrative sites to take part in 21 ideas generation workshops (*n* = 18) and yarning circles (*n* = 3). Workshops and yarning circles ran for between 45 and 180 minutes. Most participating staff were Justice Health NSW employees (81.1%) and female (86.8%). Over half were front-line staff (52.8%), and nearly two-thirds had one to ten years of experience in their current role (66.0%). Staff participant demographic characteristic data are shown in Table 2.

**Table 2:** Staff participant demographic characteristics for ideas generation workshops and yarning circles (*n* = 53).

Characteristics	*n*	%
Age, mean (SD) [range], years	40.3 (2.1) [23–62]	
Organisation		
Justice Health NSW	43	81.1
Corrective Services NSW	10	18.9
Sex		
Male	6	11.3
Female	46	86.8
Aboriginal Identity	3	5.7
Current role		
Front line	28	52.8
Non-clinical	14	26.4
People manager	11	20.8
Executive	0	0.0
Time in current role		
Less than one year	12	22.6
One to ten years	35	66.0
Greater than 10 years	6	11.3

Note: SD = Standard deviation.

Most people in prison who participated were male (70.4%), and three-fifths (63.0%) self-identified as Aboriginal. The mean (SD) age of people in prison was 41.0 (11.2) years. A majority self-reported having at least one illness (83.3%), with the most frequently reported being anxiety (53.7%) or depression (48.2%). Three-quarters were currently serving a custodial sentence (75.9%), and four-fifths had previously been in custody (83.3%). Table 3 describes the demographic characteristics of the participants in prison.

**Table 3:** People in prison participant demographic characteristics for ideas generation workshops and yarning circles (*n* = 54).

Characteristics	*n*	%
Age, mean (SD) [range], years	41.0 (11.2) [23–69]	
Male	38	70.4
Aboriginal Identity	34	63.0
English preferred language	53	98.2
Level of education		
Primary school or below	5	9.3
High school	38	70.4
Tertiary	11	20.4
Self-reported health rating		
Excellent	6	11.1
Very good	10	18.5
Good	17	31.5
Fair	15	27.8
Poor	6	11.1
Illness	45	83.3
Diabetes	6	11.1
Anxiety	29	53.7
Arthritis	8	14.8
Cardiovascular	8	14.8
Respiratory	7	13.0
Depression	26	48.2
Drug and Alcohol disorder	23	42.6
Disability	12	22.2
Other	17	31.5
Sentenced	41	75.9
Time spent in custody, mean (SD) [range], years	3.2 (4.5) [0.04-18]	
Security Classification		
Minimum	35	64.8
Medium	8	14.8
Maximum	11	20.4
Length of sentence, mean (SD) [range], years	5.7 (6.5) [0–30]	
Has previously been in custody	45	83.3
Self-reported number of times previously been in custody (range)	0-250	

Note: SD = Standard deviation.

#### Health literacy-informed action ideas arising from ideas generation workshops and yarning circles

Given that the vignettes were derived from the prison population and were co-designed with people in prison and staff^49^, participants found them to be highly realistic and often related closely to the personas depicted. This sparked rich and insightful discussions throughout the workshops and yarning circles. Vignettes featuring personas with mainly health literacy strengths (i.e., higher HLQ scale scores) or higher education levels were less familiar to participants, while those depicting individuals with greater health literacy challenges (i.e., lower HLQ scale scores) and lower education levels were widely recognised. Several people in prison identified themselves or their peers as closely resembling the individuals portrayed in these vignettes.

From the 21 workshops and yarning circles, 1119 action ideas were generated. After the removal of duplicates, 260 action ideas were identified for further analysis. Seventeen themes emerged from the analysis, which were renamed as strategies. Similar strategies were then grouped into action areas (Table 4). See Supplementary Table 1 for the full list of the 260 action ideas.

**Table 4:** Co-designed action areas, strategies and example action ideas for the New South Wales prison context.

Action area	Strategy (number of action ideas)	Example of action ideas*
1. Individualised and Continuity of Care	S1. Tailoring healthcare to individual needs and goals (23)	• Develop personalised preventative care plans.
	S2. Enabling continuity of care throughout the prison journey (11)	• Establish a Justice Health NSW community-based health clinic.
	S3. Preparing and planning for people returning to the community (10)	• Provide a 12-month program that facilitates transition back into the community.
	S4. Developing tailored programs to address specific health needs or emerging health issues (23)	• Develop and implement a disability inclusion plan for people in prison.
2. Patient and Community Engagement	S5. Harnessing the experiences and insights of people with lived experience to support others (9)	• House family members together in custody.
	S6. Enabling feedback mechanisms for people in prison to improve the system (6)	• Engage people in prison to co-design services, information and resources.
	S7. Providing education programs and meaningful activities for people in prison (17)	• Provide structured health education programs.
3. Use of Technology and Access to Information	S8. Using digital technology to provide multimodal communication and improve access to information (16)	• Enable staff to provide health services on the tablets after hours.
	S9. Developing systems to facilitate effective engagement with health services in prison (24)	• Support students to run supervised clinics.
4. Health Promotion and Preventative Care	S10. Providing health promotion and preventative care (17)	• Provide health information in wings, workplaces, and receptions areas not just in the clinic.
	S11. Performing regular screening and monitoring to track health indicators (15)	• Increase access to community screening programs.
5. Basic Needs and Resources	S12. Providing healthy food, toiletries, and medications (4)	• Provide proper toothbrushes to people in prison.
	S13. Ensuring organisations have the necessary resources, policies, and structures for effective healthcare delivery (28)	• Increase the number of Nurse Practitioners in rural centres.
6. Cultural Competency and Inclusivity	S14. Ensuring care is culturally safe and responsive for Aboriginal people (16)	• Expand the Aboriginal Chronic Care Program team.
	S15. Providing appropriate care for culturally and linguistically diverse individuals (7)	• Place people from similar cultures in the same area to provide support.
7. Collaboration and Multi-disciplinary Approach	S16. Fostering collaboration and using a multi-disciplinary approach across services (15)	• Establish a mental health liaison to coordinate with other sectors.
	S17. Building capacity of staff and attracting new talent to enhance operational performance (19)	• Raise awareness in the broader community about people in prison being part of the general population.

Note: S = Strategy; *These are examples of action ideas generated in workshops and yarning circles and do not represent all ideas. Adapted from Gill et al.^58^.

### Activity 2: Rating of co-designed action ideas by health and corrections staff

Sixty-five staff completed the survey (response rate 29.9%). Most respondents were Justice Health NSW employees (86.2%), half (47.7%) were front-line staff, and half (50.8%) had been in their current role for 1 to 5 years. One-quarter (27.7%) identified as being from a CALD background, and just under one-tenth identified as Aboriginal (7.7%). Participants also reported working across diverse NSW prison settings (Table 5).

**Table 5:** Staff participant demographic characteristics for expert rating of co-designed action ideas survey (*n* = 65).

Characteristics	*n*	%
Organisation		
Justice Health NSW	56	86.2
Corrective Services NSW	9	13.9
Length of time in current role		
less than one year	8	12.3
1–5 years	33	50.8
6–10 years	6	9.2
11–15 years	10	15.4
16–20 years	4	6.2
Greater than 20 years	4	6.2
Current role		
Front line	31	47.7
Non-clinical	12	18.5
People manager	17	26.2
Executive	5	7.7
Aboriginal Identity	5	7.7
CALD Identity	18	27.7
In what settings do you work		
Metro sites	44	67.7
Rural sites	28	43.1
Administration sites	25	38.5
Male prisons	36	55.4
Female prisons	30	46.2
Maximum security	36	55.4
Medium security	35	53.9
Minimum security	32	49.2

Note: CALD = Culturally and linguistically diverse.

Of the 260 action ideas, 73.1% (*n* = 190) were rated as moderately important, with 58 (22.3%) rated as essential and 12 (4.6%) rated by staff as not important at all^58^. None of the action ideas had been completely implemented, and 83 (31.9%) had been partially implemented. Two hundred and twenty one action ideas (85.0%) were rated as likely feasible to implement, while 18 (6.9%) were rated highly feasible. Twenty-one action ideas (8.1%) were rated as not feasible in NSW prisons. See Gill et al.^58^ for detailed survey results.

### Activity 3: Prioritisation workshop

Forty-three senior leaders responsible for the future direction and implementation of service improvements from Justice Health NSW (*n* = 37) and Corrective Services NSW (*n* = 6) attended the prioritisation workshop. Six groups—each comprising senior leaders and executives from various streams of Justice Health NSW and Corrective Services NSW—presented their prioritised action ideas to the broader group at the conclusion of the workshop.

Priority areas identified from the prioritisation workshop analysis did not directly align with the action areas stemming from the ideas generation workshops and yarning circles. The workshop’s purpose was for the senior leaders to undertake a meta synthesis, that is, to identify connections between action ideas and prioritise potential sets of action ideas for the project team and organisations to further develop, pilot test, and where appropriate, implement and evaluate.

Thematic analysis of the prioritisation workshop data revealed seven themes, which were then renamed as priority areas for Justice Health NSW and Corrective Services NSW. Each priority area has between 10 and 23 potential action ideas to support the organisations in addressing the priority. Table 6 details the priority areas, their links to the strategies identified from the ideas generation workshops and yarning circles, the number of new and refined action ideas, and examples of supporting action ideas.

**Table 6:** The seven priorities and examples of action ideas to support each priority.

Priorities (total number of actions)	Strategies the actions originated from	Number of newly generated (refined) action ideas	Example of action ideas*
1. Lived experience and support strategies (15)	S4, S5, S7, S8, S14	1 (2)	• Train people in prison to become peer workers.^#^ • Allow people in prison to bring a trusted support person to health appointments. • Facilitate virtual connections or meet-ups for people to enhance cultural connection, care and support.^
2. Communication and information sharing (16)	S6, S8, S9, S10, S15	1 (2)	• Co-design a communication strategy to enable meaningful engagements between staff and people in prison.^ • Utilise the tablets to provide programs and clinical appointments.^#^ • Inform people why they are being called up to the clinic.
3. Support throughout the prison journey (23)	S1, S2, S3, S4, S7, S8, S10, S11, S16	2 (5)	• Understand and address the issue of institutionalisation.^#^ • Reduce the number of internal transfers.^ • Implement a Multi-Disciplinary Team (MDT) review at reception or transfer between sites.
4. Access to necessities (11)	S4, S5, S7, S9, S12, S17	2 (1)	• Allow individuals to keep a limited number of Panadol tablets in their cells. • Allow simple medications, such as Panadol and Voltaren gel, to be purchased through buy-ups.^ • Utilise the sachet program to provide medication to all people in prison.^
5. Build workforce capacity and attract talent (10)	S14, S16, S17	2 (2)	• Deliver education to Justice Health NSW staff on Corrective Services NSW processes and services.^ • Understand and enhance Corrective Services NSW officers’ knowledge and awareness of holistic health.^ • Attract and increase the Aboriginal workforce across all levels of the organisations.^#^
6. Ensure models of care are fit-for-purpose (21)	S1, S4, S8, S9, S11, S13, S16	5 (4)	• Develop personalised digital medical alerts on the tablets.^ • Explore alternative models of care, such as ‘Hospital in the home’ or Artificial Intelligence triaging within our context.^ • Ensure self-referral processes are fit-for-purpose and enable timely access to care. • Develop alternative referral pathways across services.
7. Strong partnerships and shared goals (13)	S2, S9, S15, S16	1 (3)	• Extend clinic hours to enhance access to health services. • Design local solutions to facilitate access to community-based healthcare. • Develop joint local Key Performance Indicators for healthcare access.^

Note: S = Strategy; ^new action idea generated in the prioritisation workshop; ^#^action idea refined further by prioritisation workshop participants. *These are examples of action ideas and do not represent all action ideas. Adapted from Gill et al.^58^.

Across the six groups of senior leaders and executives, several common action ideas emerged. Most groups prioritised action ideas related to models of care, lived experience, communication, collaboration between organisations, digital technologies, and discharge and release planning. Some groups refined existing action ideas and proposed new ones, such as using artificial intelligence to triage the healthcare needs of people in prison. Fourteen new action ideas were suggested by the participants, with a further 19 refined throughout the prioritisation process. A complete list of prioritised action ideas is provided in Supplementary Table 2.

## Discussion

This study detailed the process of co-designing and prioritising health literacy-informed action ideas for a state-wide prison system in Australia. The co-design process leveraged the voice of people with lived experience of both being in prison or responsible for people in prison, to generate 260 health literacy-informed action ideas across seventeen strategies and seven action areas to build system-wide service improvements in the NSW prison system. Further, these health literacy-informed action ideas have been prioritised for testing, implementation and evaluation by Justice Health NSW in collaboration with Corrective Services NSW. Importantly, this guidance for service reform is based directly on the lived experiences of both people in prison and staff, reflecting what is working or could work at both the micro and macro levels. It represents their shared needs and priorities to improve access to, understanding of, and use of health information and services within the NSW prison system.

The health literacy action ideas were developed using the Ophelia process, which leverages local knowledge and expertise to co-design practical, relevant, and implementable action ideas^4,46,47^. Importantly, participants suggested action ideas that support individuals at every stage of their contact with the criminal justice system. That is, from an individual’s arrest through prison reception through to their release and reintegration into the community. This demonstrates that participants considered not only how to improve access to and use of health information and services within prison but also the broader journey through the criminal justice system. These findings demonstrate that a system-wide health literacy development approach includes continuity of care for people in prison. Such an approach may have the potential to reduce health disparities existing in communities through to a reduction of recidivism.

The utility and adaptability of the Ophelia process has been demonstrated through its application across various contexts^43,44,52^. This paper highlights how the phases and steps can be adapted for individual contexts; for example, we made two adaptations to the process. The first adaptation entailed vignettes written in first-person. Previous Ophelia projects have used third-person vignettes in the ideas generation workshops^59,60^. Following advice from our governance groups and participants, first-person vignettes were used in workshops and yarning circles after clear positive results were observed in the first three. Participants reported that the first-person vignettes were easier to relate to, which could be attributed to the persona being highly personal, enabling more profound reflections on their own experiences and perhaps feeling more inspired to help find meaningful solutions^61,62^. Secondly, unlike other studies applying the Ophelia process^5,39–45^, following thematic analysis of the workshop data, we did not organise the data into differing levels such as action ideas for individuals, community, practitioner and organisation as suggested in the Ophelia manual^47^. This organisation of the data was not undertaken due to the restrictive nature of the prison context, where people in prison have severe limitations in what they can implement, which in turn makes individual and community classification levels impractical.

Using vignettes is not new in qualitative research^63^; however, to the authors’ knowledge, this study is the first to use vignettes to portray the health literacy strengths and challenges of people in prison. Vignettes, accompanied by guiding questions, were used in workshops and yarning circles to help participants identify with and relate to the personas portrayed. Participants then drew on their personal experiences to suggest contextually relevant action ideas. Diverse participant groups across prison settings clearly recognised 20 of the 23 vignettes. However, a small number of staff did not relate to some vignettes, likely due to the specific demographics of their prison site, such as fewer people with higher health literacy profiles or differing waitlist and access challenges at that location^17^. Furthermore, people in prison commonly have lower formal education levels^12,14^, which might make vignettes with higher levels of education harder to recognise.

The large amount and variety of health literacy action ideas generated throughout the co-design workshops and yarning circles highlight the participants’ diverse perspectives. Most (95.1%) of the action ideas generated in the discussions were rated as essential or moderately important, with only 21 of the 260 action ideas rated as unfeasible by staff in the rating survey^58^. These findings show that engaging people in an authentic co-design process generates highly relevant and potentially implementable action ideas to inform system and service improvements. Moreover, the co-design process was strengthened with participants in the prioritisation workshop suggesting new and further refining previous action ideas. This process allowed a group of senior leaders to build on the insights of their staff and people in prison and then suggest action ideas that could be missing. Thus, it reassured senior leaders that the data and potential action ideas represent the organisation completely, developing ownership and trust at the highest level. This then provides a strong impetus for meaningful and potentially rapid service improvements in a large and complex group of organisations. In summary, the transparent and authentic engagement from the bottom-up and the top-down has provided a deeply informed and engaged environment for the next research phases. Building on the action ideas generated and prioritised in this study, the next steps involve co-designing the service improvements (Ophelia Phase 2) and testing, implementing, and evaluating their impact (Ophelia Phase 3)^47^ in the prison context. The outcomes, including their potential to improve health and equity, will be reported in future publications.

The findings of this study have implications for individuals undertaking health literacy-focused research in restrictive environments. This is the first application of the Ophelia process in a prison context globally, demonstrating its applicability and utility within restrictive environments. The use of bottom-up and top-down stakeholder engagement strategies has enabled strong relationships with relevant stakeholders to be built from inception. These relationships have allowed local ownership to develop. For example, organisational support from senior management for project activities and governance allowed for local staff ownership to be established. Staff ownership was strengthened through involvement in ideas generation workshops (Activity 1), the expert rating survey (Activity 2) and the prioritisation workshop (Activity 3), which enhances the usefulness and likelihood of implemented action ideas being positive and sustainable in the system.

To make progress in developing health literacy-informed action ideas across populations and communities that experience marginalisation, such as prison populations, we recommend that decision-makers consider applying the Ophelia process in combination with the HLQ. Through undertaking an initial health literacy needs assessment within their context, decision-makers can gain a deeper understanding of the strengths and challenges their population may be experiencing. This understanding enables decision-makers to co-design with relevant stakeholders, including end users, effective programs that will meet their needs. In turn, ensuring the voices of diverse stakeholders are heard, respected and leveraged, there will be motivation to improve services and systems. According to the call for action in the United Nations 2030 Sustainability Development Goals^37^, the Ophelia process provides correctional and prison health authorities with an evidence-based method to reshape services and provide equitable healthcare to people in prison. Notwithstanding the effort required to identify and deeply engage stakeholders in authentic co-design, especially in teams seeking to improve services and systems in complex settings and in resource constrained contexts like prisons, we believe the benefits of engendering local ownership of the initiative leads to effective implementation, improved sustainability, and in the long term, greater impact on health and equity.

The current study may have some limitations. Firstly, a potential limitation of this study is the selection of the prisons in which the ideas generation workshops and yarning circles were undertaken. While we believe these are typical of prisons across NSW, it is possible that selecting different prisons may have generated different sets of responses. Additionally, convenience sampling was utilised in this study which may limit the generalisability of the findings. Furthermore, people in prison were not involved in the rating survey and prioritisation of health literacy-informed action ideas. Therefore, their voice is missing, which may result in their views on what is most needed have not been captured. At the time of this study, it was not logistically possible to include all prisons or run a mixed people-in-prison-executive meeting. People who did not have adequate English comprehension or the capacity to provide informed consent were excluded from participating. Moreover, most of the staff who participated were female and Justice Health NSW staff members. Thus, the voices of male staff and Corrective Services NSW staff may have been somewhat underrepresented. The risk of this biasing the data is low, as the Ophelia process ensures all action ideas stay on the table and are never excluded due to low frequency. Moreover, the project team will engage people in prison and staff throughout the next phase of pilot testing, implementation and evaluation to minimise the impact of the potential missing voices.

## Conclusion

This study co-designed 260 health literacy-informed action ideas that have the potential to improve how people in NSW prisons access and use health information and services. The rating and prioritisation of the action ideas by staff will now inform future research, service re-design and quality improvement projects for Justice Health NSW and Corrective Services NSW. The action ideas generated represent the voices of people in NSW prisons and the staff, demonstrating that co-design can be effectively implemented within a restrictive environment. This ambitious study has advanced co-design methodologies and health literacy development for people in prison and will help shape the future of health services and policies in NSW prisons.

## Supplementary Information

Below is the link to the electronic supplementary material.

Supplementary Material 1

## Data Availability

The data generated in this study are available from the authors’ upon reasonable request.
