# Supplementary material for: Application of co-design to develop and prioritise health literacy-informed action ideas for implementation across prisons in New South Wales, Australia
Source: Sci Rep. 2025 Dec 10;15:43533. doi: 10.1038/s41598-025-27531-7 (PMC12695900; doi:10.1038/s41598-025-27531-7)
Supplement: Supplementary file 1 — Supplementary Material 1 [file 41598_2025_27531_MOESM1_ESM.pdf]

Supplementary Table 1: The seven co-designed action areas, seventeen strategies and the 260 action ideas for the New South Wales prison context.

Action area	Strategy	Action ideas
1. Individualised and Continuity of Care	S1. Tailoring healthcare to individual needs and goals	• Train staff in motivational interviewing techniques.<br>• Support people in prison to set and prioritise their health goals.<br>• Conduct intake interviews to collaboratively create comprehensive health plans, taking into account patients' past health experiences and current health needs.<br>• Put in place safe, private spaces for individuals to discuss health issues with healthcare providers.<br>• Connect individuals without family support to formal supports including official visitors or chaplains.<br>• Enable the use of advocates during healthcare appointments.<br>• Implement a system for individuals to request their preferred care worker.<br>• Obtain consent at reception to liaise with family members.<br>• Involve family members to create care plans.<br>• Establish programs to support and maintain family connections.<br>• Locate people close to home or on country for family visits.<br>• Provide workshops to people in prison for health-related goal setting and self-management.<br>• Identify opportunities and programs for people in prison to use their existing skills.<br>• Update the information pack provided at entry with current resources.<br>• Provide training to staff on the importance of care plans.<br>• Perform communication and language-based assessments at intake to develop individualised communication plans.<br>• Regularly review the placement, location and classification of individuals within the prison system.<br>• Increase access to psychology sessions.<br>• Increase the number of medical one-out cells.<br>• Standardise health service delivery across all facilities.<br>• Provide health promotion programs.<br>• Develop personalised preventative care plans.<br>• Integrate people in prisons previous medical history into current care plans.
		• Provide in-services to staff on the importance of ensuring continuity of care.

Action area	Strategy	Action ideas
	S2. Enabling continuity of care throughout the prison journey	• Adopt a throughcare model (i.e., community reintegration management and support) in the prison system.<br>• Ensure people receive their prescribed medications when transferred between centres.<br>• Establish partnerships with community-based organisations, such as Local Health Districts (LHDs) and Aboriginal Community Controlled Health Organisations (ACCHOs) to provide in-reach and out-reach services.<br>• Facilitate people in prison to have ongoing engagement with community-based health practitioners.<br>• Prescribe intake medications from the request of information (ROIs) or community medical records.<br>• Provide a support person for external appointments.<br>• Provide patients with information to take to external appointments.<br>• Expand the 'healthcare envelope project', that is sending health information with people going to external appointments.<br>• Establish a Justice Health NSW community-based health clinic.<br>• Create a Justice Health NSW team to facilitate successful transition and transfer of care to the community.
	S3. Preparing and planning for people returning to the community	• Begin discharge and release planning at reception to ensure a whole-of-custody approach.<br>• Utilise a collaborative approach to release planning, with a shared plan across Justice Health NSW and Corrective Services NSW.<br>• Ensure everyone receives release planning in a systematic way.<br>• Provide a 12-month program that facilitates transition back into the community.<br>• Provide a warm referral process (i.e., contacting a service for or with a client) for people returning to their community.<br>• Collaborate with external organisations to provide in-reach out-reach services.<br>• Justice Health NSW to provide mental health assessments for people on parole.<br>• Ensure people get their release medications by putting them into their personal property.<br>• Provide people in prison with a release pack.<br>• Provide information to people about the contents of their discharge summary.
	S4. Developing tailored	• Implement a clinical coordination model in health centres (i.e., a staff member is actively revising waiting/clinic lists, enabling patients to be seen by more than one speciality if attending the clinic).

Action area	Strategy	Action ideas
	programs to address specific health needs or emerging health issues	• Provide opportunities for people in prison to engage in music and art therapy.<br>• Develop specialised services for people with neurological needs, the aging population and other at-risk populations.<br>• Provide people in prison with mental health connection packs which include pens, envelopes, paper and a notebook.<br>• Provide support to people around domestic violence ‘regardless of whether a perpetrator or victim’.<br>• Address the issue of institutionalisation.<br>• Develop and implement a disability inclusion plan for people in prison.<br>• Replicate the Forensic Hospital’s model of family care support in prisons.<br>• Justice Health NSW establishes a health focused transition centre for people returning to the community.<br>• Justice Health NSW to take over the food provision in prison to provide better nutrition.<br>• Health runs the prisons, where Justice Health NSW runs the system, and Corrective Services NSW provide security.<br>• Increase the accountability of the organisations for example through a Royal Commission.<br>• Automate waiting list changes when people are transferred between prisons.<br>• Enable telephone voice referrals to be made to outside of hours.<br>• Take lessons from the private prisons and apply them across the greater system.<br>• Provide information to families on how to apply for accommodation and transport funding to visit people in prison.<br>• Allow for people in prison to receive care packages with essentials such as socks.<br>• Enforce mandatory savings for people in prison.<br>• Enable people in prison who are working to have improved access to sick certificates.<br>• Provide mobile phones to people at risk when leaving prison.<br>• Provide compensation pay to people in prison who get injured while working in prison.<br>• Address the poor hygiene, ventilation, and drainage issues prevalent in the prisons.<br>• Review the current dosing practices (e.g., dosing windows) for people who are on the ‘program’ to align with those in the community.
		• Establish paid peer mentor roles in prisons.

Action area	Strategy	Action ideas
2. Patient and Community Engagement	S5. Harnessing the experiences and insights of people with lived experience to support others	• Develop a program to train people in prison to become peer workers.<br>• Develop peer led education programs to help promote health and support others in custody.<br>• Enhance peer-based strategies to support people in prison such as enabling people to have a pen pal, creating a buddy system, and peer education.<br>• Engage with and use peer-based resources to provide information such as Insider News.<br>• House family members together in custody.<br>• Allow people to bring a trusted support person to health appointments.<br>• Establish paid healthcare delegate roles to help with requests and be a central point of contact.<br>• Allow qualified people in prison access to first aid supplies and defibrillators in medical emergencies.
	S6. Enabling feedback mechanisms for people in prison to improve the system	• Engage people in prison to co-design services, information and resources.<br>• Implement feedback mechanisms such as a feedback box or use a 'traffic light system' to evaluate services as people in prison receive them.<br>• Utilise the tablets to undertake surveys of people in prison.<br>• Close the communication loop and provide information regarding healthcare tests.<br>• Provide acknowledgment of receipt of a Bluey and estimated waiting time.<br>• Provide transparency and consistency for processes such as medical one out allocations and access to medical records.
	S7. Providing education programs and meaningful activities for people in prison	• Provide structured health education programs.<br>• Develop programs including exercise, back-pain, sleep, mindfulness, life skills and emotional regulation.<br>• Use Clinical Nurse Educators and other health care providers to help provide in-services and education for people in prison.<br>• Health professionals provide psychoeducation through a 12-session program that focuses on various health topics for people in prison.<br>• Provide equitable access to educational groups and programs across the system.<br>• Facilitate people in prison to complete formal education.<br>• Facilitate the development and operation of Aboriginal groups led by Aboriginal people.<br>• Provide informal and peer-based drug and alcohol programs for people in prison.<br>• All people entering custody complete a mandatory drug and alcohol program.

Action area	Strategy	Action ideas
		• Orientate people to prison and support them to establish a routine.<br>• Develop and run support groups for people newly entering prison.<br>• Provide CPR and first aid courses to people in prison.<br>• Increase the number of jobs and courses available in prison.<br>• Provide recreational activities such as sporting activities, art supplies etc.<br>• Provide art supplies to engage people in meaningful activities.<br>• Increase the variety of movies available on the tablets.<br>• Engage people in meaningful activities such as courses or employment.
3. Use of Technology and Access to Information	S8. Using digital technology to provide multimodal communication and improve access to information	• Utilise the tablets to provide programs to people in prison, such as cooking from Buy-Ups, 'Crim Fit' or a weekly health program.<br>• Use tablets to provide specific and newly developed health information to people in prison.<br>• Increase the use of multimodal information such as videos and infographics on the tablets.<br>• Provide access to Healthdirect Australia (the national virtual public health information service) on the tablets.<br>• Enhance engagement by including content from individuals with lived experience on the tablets.<br>• Provide training to people in prison on how to use the digital technology available.<br>• Roll out the step-by-step instruction booklet (already developed) for using the tablets.<br>• Prompt staff to direct people in prison to information on the tablets.<br>• Develop a peer program to teach people how to use the tablets.<br>• At reception, have the reception nurse and welfare staff teach individuals how to use the tablets and find health information.<br>• Develop digital forms on the tablets for people in prison that only have drop-down menus, including dietary, complaint and self-referral forms.<br>• Develop and resource a virtual health help desk such as a 'Facebook chat' on the tablets.<br>• Enable staff to provide health services on the tablets after hours.<br>• Develop audio and multilingual versions of health information on the tablets.<br>• Enable Justice Health NSW to use the message function on the tablet to provide feedback such as a receipt number for a Bluey or health information following an appointment.<br>• Increase access times to free phone lines on the tablets up to 10 pm.

Action area	Strategy	Action ideas
	S9. Developing systems to facilitate effective engagement with health services in prison	• Create informal ways for people in prison to engage and chat with health staff outside of the clinic.<br>• Provide outreach and mobile health clinics to worksites, yards, pods, and wings.<br>• Support students to run supervised clinics.<br>• Improve access to health staff by increasing telehealth services.<br>• Ensure medication changes are made in front of patients to provide reassurance.<br>• Separate AM and PM medications into distinct, labelled bags.<br>• Implement long versus short consults for health appointments in prisons.<br>• Require all staff to wear a visible name badge to ensure patients know their name, for example, a bright yellow badge that says hello my name is 'XX' I'm a physio.<br>• Train staff to know where relevant health information i s located.<br>• Provide people in prison with information on the basic functions of the services provided by each health discipline.<br>• Extend clinic hours to improve access to health services for people in prison.<br>• Increase the availability of adjunct and allied health therapies.<br>• Remove the 10-minute call limit for health-related phone calls.<br>• Expand the self-referral phone line to include rural centres.<br>• Ensure the self-referral processes are fit-for-purpose and enable concerns to be actioned in a timely manner.<br>• Establish clear processes that allow patients to escalate their concerns if they are not being addressed.<br>• Enable administration staff members to support people in prison to complete forms.<br>• Simplify information into easy-to-understand language for people in prison.<br>• Develop a health specific Knock Up system.<br>• Review the current Knock Up processes to ensure compliance and best practice.<br>• Inform people in prison why they are being called up to the clinic.<br>• Provide Corrective Services NSW officers a card to take to people in prison that says who is asking them to come to the clinic.<br>• Improve the culture of both organisations.<br>• Provide training to staff on the importance of maintaining professionalism.

Action area	Strategy	Action ideas
4. Health Promotion and Preventative Care	S10. Providing health promotion and preventative care	• Teach staff to use a total communication approach (e.g., including gestures, written words, pictures, sign and spoken language to support an interaction) when working with people in prison.<br>• Provide a preventive and holistic approach to health in prison.<br>• Use soft entry points (e.g., no, or very low barriers to engage) for health promotion.<br>• Provide more health information resources to people at entry and throughout their prison journey.<br>• Provide access and refer people in prison to health coaching via Healthdirect Australia (the national virtual public health information service).<br>• Develop an orientation information booklet to help people entering custody.<br>• Support people to take ownership of their health and engage in selfcare.<br>• Provide information to people in prison in different formats.<br>• Provide targeted individualised information to patients.<br>• Encourage and involve people in sporting and other competitions, such as the NSW Knockout Health Challenge.<br>• Provide targeted exercises developed by a physiotherapist.<br>• Utilise external healthcare provider information.<br>• Facilitate frequent health promotion events like Close the Gap events to encourage engagement.<br>• Provide more hands-on health promotion sessions such as making protein balls from buy-ups or dental hygiene.<br>• Engage senior managers to see the value of running health promotion events.<br>• Provide health information in wings, workplaces, and receptions areas not just in the clinic.<br>• Increase time with, and communication to people in prison.
	S11. Performing regular screening and monitoring to track health indicators	• Review the content and timings of reception screenings.<br>• Implement a Multi-Disciplinary Team at reception to include allied health professions.<br>• Complete the request of information (ROI) at police cells to enable access to necessary medications at reception to a prison.<br>• Enforce a mandatory 28-day health check-in following a request of information (ROI).<br>• Ensure we conduct specific age and high-risk health screenings for example breast, bowel, cervical and Hepatitis C screening.<br>• Increase access to community screening programs.

Action area	Strategy	Action ideas
		• Provide regular six-monthly dental and medical check-ups.<br>• Validate the health information on health records and remove inaccurate information following a yearly review.<br>• Review and improve current triage practices.<br>• Change policy to allow nurses to operate at the top of their scope to alleviate strain on the system.<br>• Increase the nursing scope of practice.<br>• Implement 'a no wrong door policy' when providing healthcare.<br>• Use student doctors and nurses to decrease current waitlists.<br>• Support and train staff to undertake opportunistic screening and care in clinics.<br>• Allow people in prison to track their own health conditions, like blood pressure and diabetes, and submit their measurement records to the clinic weekly.
5. Basic Needs and Resources	S12. Providing healthy food, toiletries, and medications	• Ensure people in prison have access to healthy food provided through CSI Food Services and Buy-Ups.<br>• Allow people in prison to keep a small number of Panadol tablets in their cell, such as four to six per day.<br>• Provide access to basic health necessities through vending machines in the prisons.<br>• Provide proper toothbrushes to people in prison.
	S13. Ensuring organisations have the necessary resources, policies, and structures for effective healthcare delivery	• Advocate to the government for increased resources to address the complex health needs of people in prison.<br>• Increase the number of Nurse Practitioners in rural centres.<br>• Review staffing models in prison to ensure adequate resources for walk-in clinics, clinical coordination, and 24-hour clinics.<br>• Use administration staff to help people in prison complete tasks that do not require a nurse, for example, pre-admission clinic questionnaire forms.<br>• Advocate for increased allied health staff and specialities.<br>• Benchmark our resources to other prisons to ensure we have equivalent resources.<br>• Justice Health NSW employs their own corrective services officers to bring patients to the clinic.<br>• Remove 'red tape' for nursing and decision making in the system.<br>• Employ an onsite manager for all streams to be accountable to.

Action area	Strategy	Action ideas
		• Upskill our managers to better support staff.<br>• Enforce Corrective Services NSW staff to commit to the hours of patient access.<br>• Implement strategies to increase the Aboriginal workforce across all levels of the organisations.<br>• Stop moving people across the state to access healthcare.<br>• Review and stop the placement of people with C classifications in maximum security centres to access appropriate healthcare as it is viewed as a form of punishment.<br>• Build local relationships at correctional centres and with community partners to find local solutions.<br>• Ensure consistency of processes and file management across centres.<br>• Increase the amount that people in prison get paid for engagement in work and other activities in prison.<br>• Move health specialities employed by Corrective Services NSW back into NSW Health.<br>• Increase the scope of practice for Corrective Services NSW psychologists to be more health focused rather than just behavioural and increase the number of sessions available.<br>• Advocate to build more new dorm style prisons and provide therapeutic environments.<br>• Allow custodial staff to touch the Corrective Services NSW tablets not just functional managers.<br>• Stop the blanket model to bans and punishment across correctional centres.<br>• Advocate for the implementation of national standards for conditions in all prisons, with a particular focus on access to medical care and supplies.<br>• Increase privacy and confidentiality for healthcare in the prison environment.<br>• Ensure access to effective pain management in prisons, like codeine for dental issues, by administering these medications under supervision.<br>• Increase access to family support though visits and phone calls.<br>• Reduce the cost of phone calls for people in prison.<br>• Give everyone in prison one free call each Sunday.
6. Cultural Competency and Inclusivity	S14. Ensuring care is culturally safe and responsive for	• Use yarning circles to engage with Aboriginal people in prison.<br>• Engage with Aboriginal Health Care Workers when working with Aboriginal people in prison.<br>• Attract more Aboriginal staff to work in the organisations.<br>• Support Aboriginal Health Workers to engage with people in pods or wings.<br>• Involve Aboriginal elders or delegates to assist with addressing issues that arise in prison.

Action area	Strategy	Action ideas
	Aboriginal people	• Develop culturally appropriate Aboriginal focused health information.<br>• Develop processes and train Aboriginal peer workers in prison to provide engagement and cultural support.<br>• Train staff about cultural nuances using case examples.<br>• Provide staff training on the cultural needs of Aboriginal people in prison.<br>• Establish partnerships and shared funding models with Local Health District's to engage Aboriginal Health Workers.<br>• Provide increased access to services like the Walama List and Circle Sentencing.<br>• Advocate to the Ministry of Health for an increase in Aboriginal positions and the expansion of the Aboriginal Chronic Care Program.<br>• Expand the Aboriginal Chronic Care Program team.<br>• Enable longer family visits and allow families to bring in food.<br>• Establish a 'Koori wing' for Aboriginal people in centres.<br>• Connect Aboriginal people with culture and art in health.
	S15. Providing appropriate care for culturally and linguistically diverse individuals	• Train staff to understand the role of culture in health and to engage in culturally appropriate ways.<br>• Increase the use of formal and informal translation services.<br>• Build partnerships with organisation to enhance connection to culture.<br>• Increase the diversity of the workforce at all levels of the organisations.<br>• Provide all information, such as referral forms and Buy-Up lists in multiple languages.<br>• Provide laminated pictures to assist non-English speaking people to communicate, e.g., toothbrushes.<br>• Place people from similar cultures in the same area to provide support.
7. Collaboration and Multi-disciplinary Approach	S16. Fostering collaboration and using a multi-disciplinary approach across services	• Facilitate better communication and information sharing with external health clinicians and organisations.<br>• Review care models to include integrated care with cross-organisational multidisciplinary teams and regular reviews.<br>• Establish care navigation or care coordination models like the Corrective Services NSW case management model.<br>• Establish clear processes for people to access the National Disability Insurance Scheme (NDIS).

Action area	Strategy	Action ideas
		• Establish shared structures, funding streams and values between Corrective Services NSW and Justice Health NSW.<br>• Review and streamline the process for using Corrective Services NSW officers to escort people in prison to the clinic.<br>• Facilitate external partners doing visits to understand our environments.<br>• Design local solutions to enable access to community-based healthcare.<br>• Develop alternative referral pathways for people in prison across services.<br>• Make specialty services readily available and visible in wings and pods.<br>• Seek input from Corrective Services NSW to improve Justice Health NSW services.<br>• Train staff on the roles of health professionals not employed by Justice Health NSW.<br>• Build better working relationships and links with universities.<br>• Increase in the number of psychologists providing services in prisons.<br>• Establish a mental health liaison to coordinate with other sectors.
	S17. Building capacity of staff and attracting new talent to enhance operational performance	• Upskill current staff in multiple specialities.<br>• Train staff to use teach back when engaging with and providing care to people in prison.<br>• Build the skill sets of staff to work with special populations (i.e., neurological disorders, personality disorders, trauma informed practices, sensitivity training).<br>• Justice Health NSW to undertake collaborative training with Corrective Services NSW particularly in rural centres.<br>• Justice Health NSW Integrated Care Services team to develop and provide release planning training to staff.<br>• Support and upskill current staff to become Nurse Practitioners.<br>• Develop training plans to upskill staff and clearly outline Justice Health NSW's future actions.<br>• Provide onsite training at rural centres by education providers and trainers travelling to rural centres.<br>• Build staff capacity in cultural competency and responsiveness.<br>• Provide education to Corrective Services NSW staff on Justice Health NSW processes and services such as the Health Problem Notification Form, scope of practice and mental health.<br>• Provide additional training to Corrective Services NSW staff working in the health clinic on patient needs, mental health first aid, advanced first aid, and managing challenging behaviours.

Action area	Strategy	Action ideas
		• Inform external agencies on the policy requirements and the appropriate health care for people in prison.<br>• Deliver lectures at universities on the healthcare needs of people in prison to enhance our profile.<br>• Raise awareness in the broader community about people in prison being part of the general population.<br>• Employ an Allied Health Educator to upskill Allied Health staff.<br>• Create rotations with Local Health Districts to increase our profile and understanding of our service and to reduce staff burnout.<br>• Undertake recruitment drives and provide financial incentives to increase staff numbers.<br>• Provide hands-on training to staff on using and navigating the Corrective Services NSW tablets.<br>• Train reception nurses to use the Corrective Services NSW tablets to instruct individuals being received into custody on how to use them.

Note: S=Strategy. Adapted from Gill et al. [58].

Supplementary Table 2: Seven priorities and the action ideas linked to the original strategy.

Priority	Action ideas	Strategy the idea originated from
1. Lived experience and support strategies.	• Train people in prison to become peer workers#	Strategy 5
	• Establish paid peer roles#	Strategy 5
	• Establish paid healthcare delegate roles to be the clinic's central point of contact	Strategy 5
	• Train Aboriginal peer workers to provide engagement and cultural support.	Strategy 14
	• Engage Aboriginal elders or delegates to assist both organisations with arising issues	Strategy 14
	• People with lived experience co-design and deliver healthcare information on tablets	Strategy 8
	• Provide access to peer-based drug and alcohol program	Strategy 7
	• Allow people in prison to bring a trusted support person to health appointments	Strategy 5
	• Replicate the Forensic Hospital’s model of family support in prisons	Strategy 1
	• Promote health and support through peer-based programs	Strategy 5
	• Run support groups for people newly entering prison	Strategy 7
	• Enhance peer-led strategies to support people in prison	Strategy 5
	• Provide mental health connection packs which include pens, envelopes, paper, and a notebook	Strategy 4
	• House family members together	Strategy 5
	• Facilitate virtual connections or meet-ups for people to enhance cultural connection, care and support^	Newly generated
2. Communication and information sharing.	• Provide more health information resources to people throughout their prison journey	Strategy 10
	• Provide visual aids to assist people in communicating their needs#	Strategy 15
	• Close the communication loop and inform people of healthcare test outcomes	Strategy 6
	• Inform people why they are being called up to the clinic	Strategy 9
	• Provide escorting staff with a card to give to people in prison stating which health professional is asking them to attend the clinic	Strategy 9
	• Direct people to information available on the tablets	Strategy 8
	• Provide tailored and newly developed health information on the tablets	Strategy 8
	• Provide access to the Healthdirect Australia website on the tablets	Strategy 8
	• Refer people to health coaching via Healthdirect Australia	Strategy 10
	• Provide healthcare information in different languages and through audio on the tablets	Strategy 8
	• Increase the availability of multimodal (e.g., oral and visual) health information on the tablets	Strategy 8

Priority	Action ideas	Strategy the idea originated from
	• Provide acknowledgment of receipt for a self-referral and expected wait time.	Strategy 6
	• Utilise the tablet message function to provide personalised health information following an appointment	Strategy 8
	• Scope the development of a virtual health help desk like a 'Facebook chat' on the tablets	Strategy 8
	• Co-design a communication strategy to enable meaningful engagements between staff and people in prison^	Newly generated
	• Utilise the tablets to provide programs and clinical appointments#	Strategy 8
3. Support throughout the prison journey.	• Obtain consent at reception to liaise with family members	Strategy 1
	• Establish programs to support and maintain family connections	Strategy 1
	• Identify stakeholders and develop processes for cross-organisational release planning#	Strategy 3
	• Provide information to people about the contents of their discharge summary	Strategy 3
	• Establish a Justice Health NSW community-based health clinic	Strategy 2
	• Advocate the government to fund and develop a 12-month community reintegration program#	Strategy 3
	• Enhance reintegration and transfer of care to the community by reviewing the current scope and function of Justice Health NSW teams^	Newly generated
	• Review the timing and content of our reception assessment screenings	Strategy 11
	• Complete the request of information (ROI) at the police cells	Strategy 11
	• Provide release packs for returning to the community	Strategy 3
	• Reduce the number of internal transfers^	Newly generated
	• Implement a Multi-Disciplinary Team (MDT) review at reception or transfer between sites#	Strategy 11 & 16
	• Perform communication and language-based assessments at reception	Strategy 1
	• Develop individualised communication plans#	Strategy 1
	• Provide mental health assessments to people on parole	Strategy 3
	• Co-design an orientation booklet for people entering custody	Strategy 10
	• Teach people in prison how to use tablets	Strategy 8
	• Engage people in meaningful activities such as courses, formal education, or employment	Strategy 7
	• Put prescribed medications in the personal property for individuals being released or transferred	Strategy 3
	• Understand and address the issue of institutionalisation#	Strategy 4
	• Establish a care navigation model for the prison journey	Strategy 16
	• Adopt a throughcare model in the prison system	Strategy 2

Priority	Action ideas	Strategy the idea originated from
4. Access to necessities.	• Train people in prison and staff to be first aid responders for medical emergencies#	Strategy 7 & 17
	• Allow qualified people access to first aid supplies and defibrillators in medical emergencies	Strategy 5
	• Provide more healthy food options through CSI food services	Strategy 12
	• Justice Health NSW takes over the nutritional oversight of CSI food services	Strategy 4
	• Allow individuals to keep a limited number of Panadol tablets in their cells	Strategy 12
	• Increase healthy food options available through buy-ups	Strategy 12
	• Allow simple medications, such as Panadol and Voltaren gel, to be purchased through buy-ups^	Newly generated
	• Provide basic health necessities through vending machines	Strategy 12
	• Provide proper toothbrushes	Strategy 12
	• Utilise the sachet program to provide medication to all people in prison^	Newly generated
	• Place morning and afternoon medications in distinct labelled bags	Strategy 9
5. Build workforce capacity and attract talent.	• Build the cultural competency and responsiveness of staff	Strategy 17
	• Deliver education to Corrective Services NSW staff on Justice Health NSW processes and services	Strategy 17
	• Deliver education to Justice Health NSW staff on Corrective Services NSW processes and services	Newly generated
	• Provide additional health training to Corrective Services NSW clinic staff	Strategy 17
	• Train staff to use gestures, written words, pictures, signs and spoken language to support interactions with people in prison	Strategy 17
	• Train staff to ask patients to report information in their own words to ensure it was understood when engaging with and providing care to people in prison#	Strategy 17
	• Deliver onsite training at rural centres	Strategy 17
	• Understand and enhance Corrective Services NSW officers' knowledge and awareness of holistic health^	Newly generated
	• Train Justice Health NSW staff on the roles of health professionals employed by Corrective Services NSW	Strategy 16
	• Attract and increase the Aboriginal workforce across all levels of the organisations#	Strategy 14
6. Ensure models of care are fit-for-purpose.	• Streamline the process for escorting people to the clinic#	Strategy 16
	• Establish guidelines to trigger the deployment of an opportunistic Multi Disciplinary Team (MDT) to strengthen and support clinical operations at a correctional centre^	Newly generated
	• Automate healthcare waiting list changes when people are transferred between prisons	Strategy 4

Priority	Action ideas	Strategy the idea originated from
	• Engage people in prison to co-design services, information, and resources	Strategy 6
	• Implement a clinical coordination model in health centres	Strategy 4
	• Advocate the government for additional resources to meet the needs of people in prison	Strategy 13
	• Expand access to adjunct and allied health therapies	Strategy 9
	• Transition health specialties currently employed by Corrective Services NSW back to NSW Health	Strategy 13
	• Expand the scope of practice of health specialities employed by Corrective Services NSW#	Strategy 13
	• Ensure self-referral processes are fit-for-purpose and enable timely access to care	Strategy 9
	• Advocate the government to fund a Justice Health NSW Aboriginal-led clinical service#	Strategy 14
	• Ensure our services are culturally safe, responsive, and trauma-informed^	Newly generated
	• Establish clear processes for individuals to access the National Disability Insurance Scheme (NDIS)	Strategy 16
	• Develop digital forms on tablets that feature only drop-down menus	Strategy 8
	• Develop personalised digital medical alerts on the tablets^	Newly generated
	• Ensure the implementation of age-specific and high-risk health screenings	Strategy 11
	• Develop alternative referral pathways across services	Strategy 16
	• Explore alternative models of care, such as 'Hospital in the home' or Artificial Intelligence triaging within our context^	Newly generated
	• Review inter-centre transfer and placement processes for healthcare access#	Strategy 1 & 13
	• Review and improve current triage practices	Strategy 11
	• Extend clinical service provision by increasing assistant roles	Newly generated
7. Strong partnerships and shared goals	• Seek Corrective Services NSW's input to enhance Justice Health NSW services	Strategy 13
	• Provide both patients and escort staff with health information to take on external appointments#	Strategy 2
	• Enhance communication and information sharing with external health clinicians and organisations	Strategy 16
	• Create shared structures, funding streams and values between Corrective Services NSW and Justice Health NSW	Strategy 16
	• Establish partnerships and shared funding models with Local Health District's to engage Aboriginal Health Workers and other health professions	Strategy 14
	• Establish partnerships with community-based organisations to provide in-reach and out-reach services	Strategy 2
	• Establish cross-organisational Multi-Disciplinary Teams at centres to facilitate regular reviews#	Strategy 16
	• Design local solutions to facilitate access to community-based healthcare	Strategy 16

Priority	Action ideas	Strategy the idea originated from
	• Increase our organisation's profile and raise awareness of the healthcare needs of people in prison#	Strategy 17
	• Facilitate visits to increase external partners understanding of our environment	Strategy 16
	• Strengthen connection to culture through partnerships with local organisations	Strategy 15
	• Develop joint local Key Performance Indicators for healthcare access^	Newly generated
	• Extend clinic hours to enhance access to health services	Strategy 9

Note: ^new action generated in the prioritisation workshop; #action refined further by prioritisation workshop participants. Adapted from Gill et al. [58].
